# Primary del 17 chronic lymphocytic leukaemia lymphocytes are hypersensitive to dasatinib *in vitro*

**DOI:** 10.1111/j.1365-2141.2009.07814.x

**Published:** 2009-09-02

**Authors:** Lilian Amrein, Lawrence Panasci, Spencer B Gibson, James B Johnston, Denis Soulières, Raquel Aloyz

**Affiliations:** 1 Lady Davis Institute for Medical Research-Cancer Segal Center, Sir M.B. Davis-Jewish General HospitalMontreal, QC; 2 Montreal Center for Experimental Therapeutics, McGill UniversityMontreal, QC; 3 Manitoba Institute of Cell Biology, Cancer Care ManitobaWinnipeg, MB; 4 Department of Medicine, Service of Hematology and Medical Oncology, Centre Hospitalier de l’Université de Montréal and Centre de Recherche du CHUMSherbrooke East, Montreal, QC, Canada E-mail: raloyz@yahoo.com

Chronic lymphocytic leukaemia (CLL) is characterized by the accumulation of mature quiescent B-lymphocytes in the G0/G1 phase of the cell cycle. B-lymphocyte accumulation is likely to be a consequence of an undefined defect in the apoptotic machinery rather than an increased proliferation of leukaemic cells (Hamblin & Oscier, 1997). The prolonged survival of CLL lymphocytes has also been linked to deregulated expression and/or activity of related non receptor tyrosine kinases including members of the *SRC* family kinases (SFK) and *ABL1*. Inhibition of either c-abl (Aloyz *et al*, 2004) or SFK (Contri *et al*, 2005) results in CLL lymphocyte death *in vitro*. Previous results from our laboratory suggest that dasatinib cytotoxicity in CLL lymphocytes is associated with c-abl rather than SFK inhibition (Amrein *et al*, 2008a). Recent results of phase I–II clinical trials using dasatinib in CLL suggest that the drug might be beneficial in only a small subset (≤10%) of previously treated patients (Amrein *et al*, 2008b). In agreement with these clinical results, we have recently reported that dasatinib is cytotoxic to primary CLL lymphocytes *in vitro* but mainly at clinically unobtainable concentrations (Amrein *et al*, 2008a). Although dasatinib resistance was associated with the basal expression of c-abl, our study suggested that wild type *TP53* is important in CLL lymphocyte homeostasis and/or survival in the presence of dasatinib (Amrein *et al*, 2008a). To test this hypothesis we assessed: (i) dasatinib cytotoxicity in p53 proficient (wild type) CLL lymphocytes treated with dasatinib in the presence or absence of pifithrin*-*α, a small molecule inhibitor of p53 transcriptional activity (Steele *et al*, 2008) and 2) dasatinib cytotoxicity in primary CLL lymphocytes with impaired *TP53* signalling from patients diagnosed with del 17p13.1. In the eleven samples we verified the functionality of p53 signalling as described before by examining changes in p53 and its downstream target p21Cip1 (p21) after treatment with chlorambucil (Willmore *et al*, 2008). Briefly, the lymphocytes were treated with equivalent cytotoxic concentrations of chlorambucil (50% inhibitory concentrations [IC_50_’s]) for 24 h and induced p53 and p21 protein levels were monitored by Western blotting. As expected p53 and p21 protein levels were induced by chlorambucil only in *TP53* wild type CLL lymphocytes (data not shown).

As previously reported, dasatinib IC_50_s *in vitro* in CLL lymphocytes expressing wild type *TP53* were not in the clinically attainable range (mean value of 30 μmol/l, Table I) (Amrein *et al*, 2008a). In contrast, the dasatinib IC_50_s in del 17p13.1 lymphocytes were significantly lower (up to 100 times) than in the wild type *TP53* lymphocytes. Moreover, in agreement with previous reports demonstrating that del 17p13.1 is associated with chemoresistance in CLL lymphocytes, we found that del 17p13.1 lymphocytes were significantly more resistant to chlorambucil that *TP53* wild type CLL lymphocytes [Table I, Fig 1A (Zenz *et al*, 2008)].

**Table I:** Clinical characteristic of the patients and IC_50_ concentrations of dasatinib and chlorambucil in primary CLL-lymphocytes *in vitro*.

Patient	Deletion 17 Deletion 11	RAI stage	Previous treatment	Dasatinib (μmol/l)	CLB (μmol/l)
1	Negative	I	CLB	0·8	17·3
2	Negative	0	FLU	30·6 (2·4)	23·0
3	N.D.	III	CLB	7·2	30·0
4	Negative	0	No	0·1	41·0
5	Negative	0	No	36·0 (27·0)	18·6
6	N.D.	III	CLB	28·2	10·9
7	Negative	II	CLB	40·0 (40·0)	24·9
8	Del 17p13.1 (86%) Del 11q22–23 (N.D.)	II	CLB	0·27	36·7
9	Del 17p13.1 (94%) Del 11q22–23 (5%)	I	CLB/FLU/Rituximab	0·01	44·7
10	Del 17p13.1 (79%) Del 1q22–231 (76%)	I	No	0·01	100
11	Del 17p13.1 (33%) Del 11q22–23 (10%)	II	CLB	0·17	100

Primary CLL lymphocytes, isolated and plated as described above, were incubated in the presence of dasatinib or chlorambucil (CLB). The IC_50_ concentrations are expressed in μmol/l and determined after 72 h incubation *in vitro* using the MTT (3-(4,5-Dimethylthiazol-2-yl)-2,5-Diphenyltetrazolium Bromide) assay as described (Amrein *et al*, 2008a). The numbers in parentheses indicate the dasatinib IC_50_ in the presence of 25 μmol/l pifithrin-α. The percentage of lymphocytes with del 17 and del 11 in each samples are indicated. Samples displaying percentages above 4·8% and 6·6% for del 17 and del 11 respectively were considered positive.

Negative percentage lower than the cut-off point.

N.D. not determined; CLB, chlorambucil.

**Fig 1:**
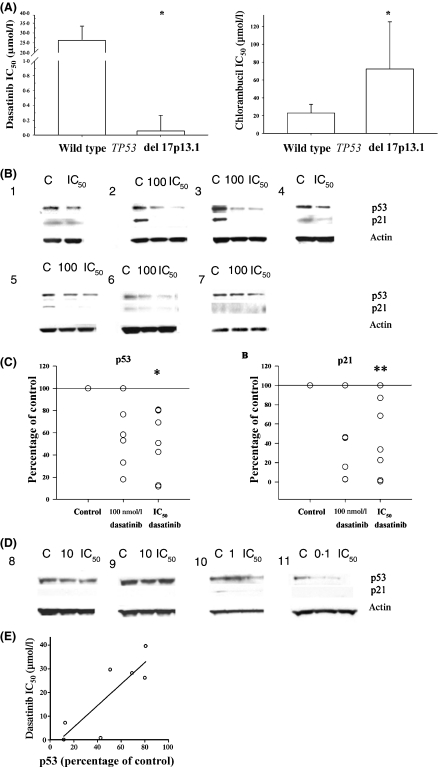
Dasatinib decreases p53 basal expression levels in primary CLL lymphocytes expressing wild type p53. Dasatinib and chlorambucil IC_50_s were significantly different between CLL lymphocytes expressing wild type *TP53* or del 17 (*P = 0.012). The bars represent the median values and 95% confidence intervals (CI 95%). (A). The lymphocytes of 11 CLL lymphocyte patients were treated for 24 h with vehicle (dimethyl sulphoxide), dasatinib 100 nmol/l or the IC_50_ concentration as shown in Table I. Protein extracts were obtained as described before and 50 μg of proteins for each sample were resolved by sodium dodecyl sulphate poyacrylamide gel electrophoresis. p53 and p21 protein levels were assessed by Western blot using specific antibodies (Amrein *et al*, 2008a). The signals obtained in *TP53* wild type lymphocytes (B) were analysed using National Institutes of Health -Scion image and normalized to actin, p53 or p21 levels (*y*-axis) and are expressed as the percentage of vehicle treated lymphocytes (control) value ([OD value/control OD value]x 100) vis à vis the treatments indicated in the *x*-axis; * and ** indicates significance *P* = 0·003 and *P* = 0·004 respectively (C). Dasatinib-induced changes in p53 levels and p21 signal were not detected in protein extracts from del 17p13·1 CLL lymphocytes (D). Dasatinib IC_50_s correlate with the percentage of residual p53 protein levels (in respect to vehicle treated lymphocytes) after dasatinib treatment, *r* = 0·82, *P* = 0·02 (E). Two-sided tests with α-value of 0·05 were used. Correlations between the data were assessed using the Spearman test. All tests were performed using SigmaStat software.

In available wild type *TP53* CLL samples, pifithrin*-α* sensitized the lymphocytes of 2 out 3 patients (1·3 and 12-fold) (Table I). Importantly, pifithrin-*α* was not toxic to CLL lymphocytes when used alone. Treatment with dasatinib for twenty four hours resulted in a dose dependent reduction of p53 and p21 basal expression levels in the lymphocytes of patients expressing wild type *TP53* (Fig 1B, C). In contrast, p53 levels were not affected in del 17 lymphocytes. Importantly, p21 was not detected in del 17p13.1 lymphocytes suggesting that *TP53* is not functional (Fig 1D). These results are in agreement with previous reports suggesting that in the majority of CLL patients with malignant lymphocytes displaying del 17p13.1, the remaining *TP53* allele is mutated (Zenz *et al*, 2008).

In addition, we found that dasatinib resistance (higher IC_50_) in CLL lymphocytes expressing wild type *TP53* correlated with residual p53 protein levels (in respect to control) after dasatinib treatment (*r* = 0·8, *P* = 0·02, Fig 1E). As *ATM* is a key regulator of p53 functionality, we assessed del 11q22-23 status in del 17p13.1 lymphocytes (Table I) (Pettitt *et al*, 2001). Two of the three del 17p13.1 samples tested were positive for del 11q22–23. Although del 17p13.1 lymphocytes were hypersensitive when compared to p53 proficient lymphocytes, we did not find a correlation between the percentage of del 17p13.1 or del 11q22–23 in the lymphocytes and the IC_50_ of dasatinib. Studies regarding the role of p53 signalling (and its regulators e.g. ATM) in dasatinib sensitivity in a larger cohort of CLL samples should be informative.

Taken together, our results suggest that p53 is important to maintain CLL lymphocyte homeostasis following exposure to dasatinib and suggest that dasatinib may be effective to treat del 17p13.1 CLL patients. The recent report of an excellent clinical response to dasatinib of a CLL patient with lymphocytes displaying del 17p13.1 supports this hypothesis (Pitini *et al*, 2009).

## References

[b1] Aloyz R, Grzywacz K, Xu ZY, Loignon M, Alaoui-Jamali MA, Panasci L (2004). Imatinib sensitizes CLL lymphocytes to chlorambucil. Leukemia.

[b2] Amrein L, Hernandez TA, Ferrario C, Johnston J, Gibson SB, Panasci L, Aloyz R (2008a). Dasatinib sensitizes primary chronic lymphocytic leukaemia lymphocytes to chlorambucil and fludarabine in vitro. British Journal of Haematology.

[b3] Amrein PC, Attar E, Takvorian T, Hochberg E, Ballen KK, Leahy KM, Neuberg D, Brown JR (2008b). Dasatinib Has Activity in Relapsed/Refractory Chronic Lymphocytic Leukemia (CLL/SLL), a Phase II Trial. Blood.

[b4] Contri A, Brunati AM, Trentin L, Cabrelle A, Miorin M, Cesaro L, Pinna LA, Zambello R, Semenzato G, Donella-Deana A (2005). Chronic lymphocytic leukemia B cells contain anomalous Lyn tyrosine kinase, a putative contribution to defective apoptosis. Journal of Clinical Investigation.

[b5] Hamblin TJ, Oscier DG (1997). Chronic lymphocytic leukaemia: the nature of the leukaemic cell. Blood Reviews.

[b6] Pettitt AR, Sherrington PD, Stewart G, Cawley JC, Taylor AMR, Stankovic T (2001). p53 dysfunction in B-cell chronic lymphocytic leukemia: inactivation of ATM as an alternative to TP53 mutation. Blood.

[b7] Pitini V, Arrigo C, Altavilla G (2009). Dasatinib induces a response in chronic lymphocytic leukemia. Blood.

[b8] Steele AJ, Prentice AG, Hoffbrand AV, Yogashangary BC, Hart SM, Nacheva EP, Howard-Reeves JD, Duke VM, Kottaridis PD, Cwynarski K, Vassilev LT, Wickremasinghe RG (2008). p53-mediated apoptosis of CLL cells: evidence for a transcription-independent mechanism. Blood.

[b9] Willmore E, Elliott SL, Mainou-Fowler T, Summerfield GP, Jackson GH, O’Neill F, Lowe C, Carter A, Harris R, Pettitt AR, Cano-Soumillac C, Griffin RJ, Cowell IG, Austin CA, Durkacz BW (2008). DNA-dependent protein kinase is a therapeutic target and an indicator of poor prognosis in B-cell chronic lymphocytic leukemia. Clinical Cancer Research.

[b10] Zenz T, Benner A, Dohner H, Stilgenbauer S (2008). Chronic lymphocytic leukemia and treatment resistance in cancer: the role of the p53 pathway. Cell Cycle.

